# Medical students’ education on organ donation and its evaluation during six consecutive years: results of a voluntary, anonymous educational intervention study

**DOI:** 10.1186/s40001-015-0116-6

**Published:** 2015-03-12

**Authors:** Sonia Radunz, Tamás Benkö, Sabrina Stern, Fuat H Saner, Andreas Paul, Gernot M Kaiser

**Affiliations:** Department of General, Visceral and Transplantation Surgery, University Hospital Essen, Hufelandstr. 55, 45147 Essen, Germany

**Keywords:** Education, Intervention study, Medical students, Organ donation, Organ donor card, Organ shortage

## Abstract

**Background:**

One of the main reasons for organ shortage is insufficient education on organ donation. Knowledgeable medical students could share the information with friends and families resulting in a positive attitude to organ donation of the general public.

**Methods:**

During six consecutive years (2009 to 2014), we conducted a voluntary, anonymous educational intervention study on organ donation among fourth year medical students in the course of the main surgery lecture at the University of Essen, Germany.

**Results:**

Questionnaires of 383 students were analyzed. Prior to the specific lecture on organ donation, 64% of the students carried a signed organ donor card with the intention to donate. Further information regarding organ donation was required by 37% of the students. The request for further information was statistically significantly higher among students without a donor card compared to organ donor card carriers (*P* < 0.0001). After the lecture, the number of students requiring further information decreased statistically significantly to 19% (*P* < 0.0001).

**Conclusions:**

Already a 45-minute lecture for fourth year medical students significantly decreases their request for further information on organ donation and improves their attitude to organ donation. Continued training on organ donation will help medical students to become disseminators for this important topic in our society.

## Background

Persisting donor organ shortage remains a limiting factor for transplantation and has to be considered a serious worldwide health problem. Regardless of extensive efforts to increase deceased organ donation, the refusal rate for organ donation remains high and the supply of donor organs does not meet the growing demand for transplantation [1].

Lack of educational programs on organ donation and transplantation has been pointed out before as one of the main reasons for organ shortage [2]. Refusal to consent to organ donation is often based on prejudices, and many potential donors are lost due to limited information and communication. Rey *et al.* identified an information deficit in young people, that is, high school seniors, as one of the main causes for inadequate acceptance of organ donation [3]. Medical students and even residents and fellows also possess limited knowledge about organ donation which might be attributed to the way of training [4-6]. A sustained knowledge may meet misconceptions about organ donation and transplantation, especially in times of widely published individual cases of misconduct in the organ transplant allocation system as recently occurred in Germany.

Successful organ procurement will continue to rely on a favorable attitude of health care professionals. Thus, educating health care professionals and people as a whole on organ donation remains essential to encourage organ donation. D’Alessandro *et al.* demonstrated that college students who are registered organ donors are more likely to serve as advocates for organ donation and help increase awareness and knowledge of others [7]. Gauher *et al.* found that medical education specifically had an important effect on shaping attitudes toward organ donation [8]. Communication with others about organ donation increases the willingness of individuals to have favorable attitudes to being an organ donor [9]. Knowledgeable medical students could pass on the information and discuss the topics with their families and friends, resulting in a positive attitude to organ donation of the general public [10]. To increase the number of potential organ donors, structured education, assessment of training deficits, and evaluation of performance therefore need to be intensified.

The objective of this study was to evaluate the impact of a specific lecture on information needs and attitude of medical students concerning organ donation. Medical students are potential resources for communicating knowledge about organ donation to the public and may become opinion leaders. It is hypothesized that education can significantly expand their perceived knowledge and enhance their attitude to organ donation.

## Methods

During six consecutive years (2009 to 2014), we conducted a voluntary, anonymous educational intervention study on organ donation at the University of Essen, Germany. The study was approved by the local ethics committee (Ethik-Kommission der Medizinischen Fakultät der Universität Duisburg-Essen) and was conducted in accordance with the Helsinki Declaration of 1975, as revised in 2008. The intervention study focused on fourth year medical students attending the main surgery lecture which comprises five semester hours. The study occurred over 6 years, that is, only fourth year students over the past 6 years were surveyed, and all study participants were fourth year students.

The study design was selected to evaluate the impact of a lecture on information level and attitude to organ donation. Intervention was composed of a baseline questionnaire, a specific lesson by a trained transplant surgeon and a second questionnaire. The type of questions asked was structured dichotomous. For demographics, age and sex were recorded. During the lecture, organ donor cards were handed out to the students.

At our university, the main surgery lecture is the venue where organ donation is typically introduced. This 45-minute lecture has now become an integral part of the main surgery lecture for fourth year students. During this specific lecture, students are informed on legal aspects of organ donation and transplantation, transplant waiting lists, brain death criteria and diagnostics, intensive care treatment of potential organ donors, the difficult care for relatives of potential organ donors, and the organ donation process.

Prior to the lecture, attitude to organ donation was assessed by determining whether the students carried a signed organ donor card with the decision to donate organs or whether they could imagine carrying an organ donor card in the future. Training deficits were evaluated through questions asking whether students were receiving first time information on organ donation and whether they wished for more information concerning organ donation. Societal and ethical factors that may affect students’ attitudes toward organ donation were not considered. After the lecture, attitude to organ donation was assessed again and persisting training deficits were evaluated.

Data collection and statistical analysis were performed using Microsoft Excel 2013 (Microsoft Corporation, Redmond, WA, USA) and GraphPad Prism version 5.00 for Windows (GraphPad Software, San Diego, CA, USA). Data are expressed as percentages and absolute numbers if not stated otherwise. Statistical analysis was performed using Fisher’s exact test or the chi-square test as appropriate, with a *P* value of 0.05 or less taken to demonstrate statistical significance. To compare differences over time, Student’s *t*-test was performed.

## Results

Questionnaires of 383 medical students were analyzed. Of the respondents, 32% (*n* = 122) were male and 68% (*n* = 261) were female. The respondents had a mean age of 24 ± 1 years.

Prior to the specific lecture on organ donation, 64% (*n* = 244) of the students carried a signed organ donor card certifying their willingness to donate after death. An additional 26% (*n* = 100) stated they might carry an organ donor card in the future. When comparing male and female students, more female students were already carrying a donor card (66% vs. 59%) and even more intended to do so in the future (26% vs. 15%). This did not, however, reach statistical significance.

**Figure 1:**
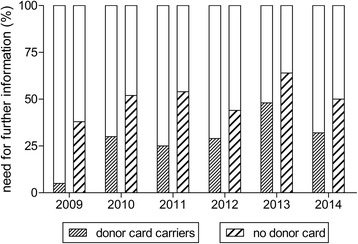
**Statistically significantly higher request for further information regarding organ donation among students without a donor card (** ***P*** **< 0.0001).**

Only 6% (*n* = 21) of the medical students received first-time information regarding organ donation during the lecture. Further information regarding organ donation was required by 37% (*n* = 142) of the students. The request for further information was statistically significantly higher among students without a donor card compared to organ donor card carriers (*P* < 0.0001) as seen in Figure 1. The highest request for further information (57%) was among female students without a donor card.

**Figure 2:**
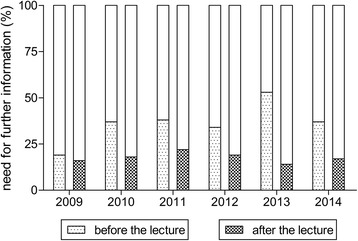
**Statistically significant decrease in request for further information regarding organ donation after the lecture (** ***P*** **< 0.0001).**

**Figure 3:**
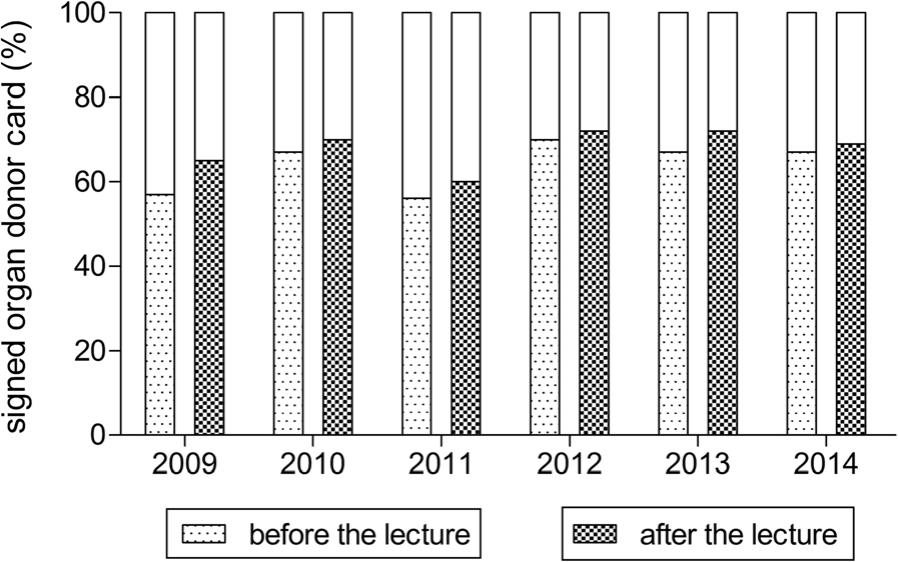
**Rate of organ donor card carriers before and after the lecture (** ***P*** **0.32).**

After the lecture, the number of students requiring further information regarding organ donation decreased statistically significantly to 19% (*n* = 71) of the students (*P* < 0.0001) as seen in Figure 2. The rate of organ donor card carriers increased to 67% (*n* = 258) as seen in Figure 3. However, the observed increase did not reach statistical significance. Additional 24% (*n* = 89) stated they might carry an organ donor card in the future.

During the six consecutive years in which the lecture was given, there were no statistically significant changes in organ donor card carriers, in the number of respondents receiving first-time information regarding organ donation, and in the number of respondents requiring further information regarding organ donation when looking separately at the annual results.

There were no differences in the answers due to the age of the participants as all participants are roughly the same age. When looking at gender differences, there were also no statistically significant differences.

## Discussion

Worldwide, mortality on transplant waiting lists has been increasing because of persisting donor organ shortage even though the transplant community has adopted various strategies to expand the donor pool, such as accepting extended criteria donors or living donors [11,12]. Therefore, organ donation and transplantation remain dependent on support and a favorable attitude from health care professionals and the general public. Improvements in knowledge about organ donation and consequently positive attitudes to donation after death may lead to significantly higher numbers of potential donors and a significantly increased consent rate.

The positive effect of educational programs has been demonstrated [13-15]. Irving *et al.* proved that factors having the greatest influence on individuals’ donation decision included knowledge and information level [16]. In our study, too, respondents who considered themselves sufficiently knowledgeable about organ donation were more often carriers of an organ donor card. Specific knowledge about organ donation apparently reduces misconceptions about organ donation and transplantation and seems therefore to ensure a positive attitude to organ donation.

This is especially important in countries with an opt-in solution, for example Germany, where the information of the public may be of even higher importance as compared to countries, for example Spain or Austria, with an opt-out solution. A recent scandal of manipulating the organ transplant allocation system substantially undermined the public confidence in the organ donor system, and donation rates decreased considerably in Germany. Despite these allegations, we demonstrated a continuously high rate of organ donor card carriers among medical students as they seem to be well-informed and not influenced as much as the general public by mass media coverage.

The majority of health care workers are already favorable toward organ donation, and being a doctor is highly predictive of willingness to donate an organ [17]. Nevertheless, there still is a substantial lack of specific knowledge about organ donation in society as a whole as well as among health care professionals [18]. There remains a need for greater public awareness of organ donation and for available information on organ donation. Effective educational programs must continue to be the main source of information regarding organ donation. They should give precise information on brain death, its diagnosis, and organ allocation, and thus raise public awareness and commitment to organ donation [19,20].

Efforts must be aimed at involving medical schools in continuous education on organ donation [2]. In an online survey distributed through social networks in Italy, Cucchetti *et al.* found 81.9% of participants thinking schools should provide education regarding donation and 68.5% thinking that family doctors should provide such information. Specific knowledge about organ donation principles was the main factor associated with a positive attitude toward donation [21].

Any measures dealing with the problem of organ shortage must therefore include educating medical students. Health care professionals should provide information regarding organ donation and guide families and potential donors in any aspect of organ donation and transplantation. Extended professional knowledge of and training on organ donation and transplantation will relate to a more positive attitude to organ donation.

Can a 45-minute lecture for fourth year students really be expected to change their attitude regarding organ donation? Many educational interventions are considered too brief and too episodic to have much impact. Yet, a 1-hour training intervention among clerks at the Department of Motor Vehicles (DMV) significantly increased their knowledge, attitude, and behavioral intention toward organ donation, resulting in higher organ donor registration rates [22]. We chose our study design of one lecture only and immediately before and afterwards distributed questionnaires to eliminate any external factors. Participants were asked for perceived knowledge and self-reported attitude so that the study design itself could not influence results by way of sensitizing participants beforehand.

Nevertheless, our lecture in its current form may not be effective enough as yet and it might have to be modified. The specific lecture did impact a significant change in the students’ perceived knowledge of organ donation, but the students’ decision to register as an organ donor did not increase significantly. These results are comparable to a study by McGlade *et al.* After a much more intense program of 33 hours on organ donation, student nurses also demonstrated an increased willingness to donate and a rising number actually registering, but these numbers did not reach statistical significance either [23]. In our study, the number of donor card carriers before the lecture was much higher compared to the general public suggesting a better informed group resulting in already high registration rates [24]. Over the course of 6 years, results were highly reproducible for medical students at our institution but may not be generalized to other universities.

Well-directed programs need to be applied to continuously alert young adults to the topic of organ donation and to raise awareness of organ shortage. In an extensive study that developed a comprehensive model on the relative importance of cognitive, attitudinal, and social dimensions on the organ donor registration process, D’Alessandro *et al.* demonstrated that social-based communications had the second greatest impact on support of organ donation and actual donor registration [7]. Students have a very high usage of social media and self-reported a strong likelihood to use personal and electronic communication to access information about social causes and the willingness to distribute user-generated content in support of a cause. Cameron *et al.* also reported that novel applications of social media may prove effective in increasing organ donation rates [25]. Heuer *et al.* showed that the use of e-mail communication is an important tool to inform the public and facilitate the documentation of a decision about organ donation [26]. The insertion of accurate information on organ donation in a television drama was also found effective in promoting positive discussion about organ donation [27]. More research is needed to find ways to merge personal and media campaigns.

## Conclusion

Our results support the conclusion that already only one brief intervention can significantly increase perceived knowledge of organ donation and positively influence attitude to organ donation among fourth year medical students. Intensive international effort in structured education on organ donation and further evaluation of the effectiveness of content delivery is imperative. This effort could increase the rate of organ donation and transplantation if measures are implemented in every medical school accessible for all medical students.
